# Sewer Gases

**Published:** 1877-11

**Authors:** 


### SEWER GASES.

Prof. Frankland has made another contribution to sanitary science by stating his conclusion, after repeated experiments, that sewage, in flowing through a sewer, however unpleasant the odor may be in the locality, cannot be sufficiently agitated to impregnate the circumambient air with suspended particles. But if sewage becomes stagnant, fermentation ensues, and the bursting of myriads of minute bubbles throws into the air particles of zymotic matter. If, therefore, sewage is constantly passing at a fair rate through the sewers, the air therefrom is comparatively harmless; but if it be allowed to remain long enough to putrify, danger to health may arise.—*London Lancet*, July, 1877.

